# Social Engagement and Well-Being Among Chinese Older Adults

**DOI:** 10.1093/geroni/igab046.1132

**Published:** 2021-12-17

**Authors:** Wei Zhang, Bei Wu, Yan Yan Wu

## Abstract

Social engagement is increasingly recognized as a protective factor to promote healthy aging. This symposium provides new findings on social participation and social isolation in relation to individuals’ health and well-being among the Chinese populations. Using the 2002¬¬¬¬¬¬¬¬¬¬¬¬¬¬¬¬¬¬¬¬¬¬¬¬¬¬¬¬¬¬¬¬¬¬¬¬¬–2018 Chinese Longitudinal Healthy Longevity Survey, the first study examined the trends of leisure activity engagement among young-old adults aged 65–74 in China over a 16-year period. Findings revealed a general downward trend of engaging in any form of social leisure activity and upward trends for engaging in home-bound and solitary leisure activities. Similarly, the second study examined social participation patterns and individual factors associated with these patterns using three nationally representative data in China, UK, and US. Their findings highlighted several underlying participation patterns across these nations as well as differences in how socio-demographics were associated with these patterns. Using data collected among Chinese older adults in Hawaii, the third study examined the associations of social isolation with psychological well-being. Results showed that social isolation was positively related to psychological distress, and negatively related to life satisfaction and happiness. These associations were partially mediated by resilience. Their findings revealed the detrimental health effects of social isolation. Using the same dataset in Hawaii, the last study examined the associations between neighbourhood conditions and psychological well-being for Chinese older adults. Their findings revealed that both physical and social neighbourhood conditions were associated with psychological well-being, particularly for foreign-born older adults, and psychological resources such as self-management abilities could mediate the associations.

